# The process of nitrogen-adaptation root endophytic bacterial rather than phosphorus-adaptation fungal subcommunities construction unveiled the tomato yield improvement under long-term fertilization

**DOI:** 10.3389/fmicb.2024.1487323

**Published:** 2025-01-17

**Authors:** Xiaoxia Li, Muhammad Awais, Shuang Wang, Zhu Zhang, Shuning Zhao, Yufeng Liu, Zhouping Sun, Hongdan Fu, Tianlai Li

**Affiliations:** ^1^College of Horticulture, Shenyang Agricultural University, Shenyang, China; ^2^Key Laboratory of Protected Horticulture of Education Ministry and Liaoning Province, Shenyang, China; ^3^National and Local Joint Engineering Research Center of Northern Horticultural Facilities Design and Application Technology, Shenyang, China; ^4^College of Agriculture, Eastern Liaoning University, Dandong, Liaoning, China

**Keywords:** endophyte, community assembly, abundant microbial taxa, rare microbial taxa, solar greenhouse

## Abstract

Interactions between endophytes (endophytic bacteria and fungi) and plants are crucial in maintaining crop fitness in agricultural systems, particularly in relation to abundant and rare subcommunities involved in community construction. However, the influence of long-term fertilization on heterogeneous rhizosphere nitrogen and phosphorus environments and how these conditions affect the key subcommunities of root endophytes and their community assembly mechanisms remain unclear. We studied the 26th year of a field experiment conducted in a greenhouse with varying levels of nitrogen and phosphorus (CKP0, CKP1, CNP0, CNP1, ONP0, and ONP1) to assess the composition of tomato root endophytes and their impact on yield. We employed 16S rRNA and fungal ITS region amplicon sequencing to investigate the assembly mechanisms of abundant and rare endophytic subcommunities, network correlations, core subcommunity structures, and key species that enhance crop yield. The results indicated that organic manure and phosphorus fertilizers significantly increased the rhizosphere soil nitrogen content, phosphorus content, and phosphorus availability (labile P, moderately labile P, and non-labile P). These fertilizers also significantly affected the composition (based on Bray-Curtis distance) and community assembly processes (βNTI) of endophytic microbial subcommunities. The assembly of both bacterial and fungal subcommunities was primarily governed by dispersal limitation, with community structures being significantly regulated by the content of rhizosphere soil available nitrogen (AN) and moderately labile P (MLP). Rare bacterial and fungal subcommunities complemented the ecological niches of abundant subcommunities in the co-occurrence network, supporting community functions and enhancing network stability. Nitrogen-adapting abundant and rare bacterial subcommunities provided a stronger predictive correlation for tomato yield than phosphorus-adapting fungal subcommunities. Additionally, three core genera of rare endophytic bacteria such as *Arthrobacter*, *Microbacterium*, and *Sphingobium* were identified as potentially involved in improving crop yield improvement. These findings revealed the distinct assembly mechanisms of endophytic microbial subcommunities affected by fertilization, enhancing our understanding of better management practices and controlling endophytes to improve crop yield in intensive agricultural ecosystems.

## Graphical abstract

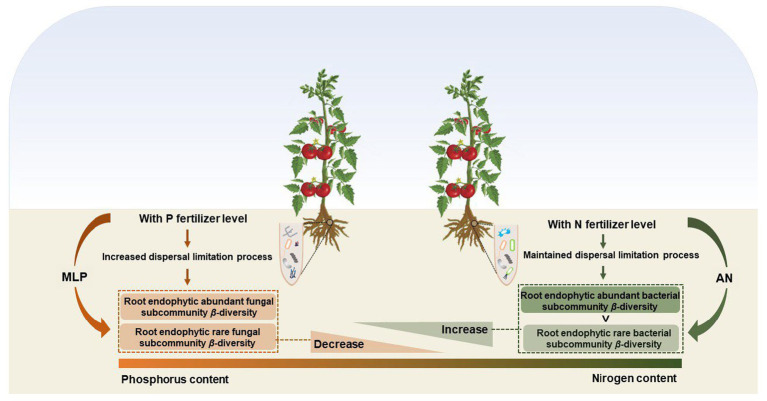

## 1 Introduction

Endophytes are a group of living microorganisms that can successfully colonize various plant tissues without causing disease symptoms (Adeleke and Babalola, 2021; Santoyo et al., 2016). Plants develop complex coexistence and mutualistic relationships with various endophytes (Battu and Ulaganathan, 2020; Harman and Uphoff, 2019) due to the essential functions provided by these endophytes, such as nutrient acquisition (Bolan, 1991; Burragoni and Jeon, 2021; Xie et al., 2024), stress tolerance (Ali et al., 2014; Battu and Ulaganathan, 2020; Harman and Uphoff, 2019; Zhang et al., 2024), and disease suppression (Adeleke et al., 2021; Card et al., 2016; Omomowo and Babalola, 2019; Sena et al., 2024). The mutualistic benefits between plants and endophytes also depend on the genetic and environmental conditions of the host (Meng et al., 2022). In particular, plant root endophytes are inherited through host genes and successfully colonize after filtrating through rhizosphere microorganisms (Meng et al., 2022; Xie et al., 2024). Hence, plant root endophytes exhibit survival strategies similar to those of rhizosphere soil microorganisms, offering significant benefits for healthy plant growth and improved yield (Guo et al., 2024; Harman and Uphoff, 2019). Root endophyte symbiosis, which improves plant adaptability under stress conditions, is increasingly important. Notably, some root endophytes have developed strategies to adapt to the unique soil dynamic environments (Beltran-Garcia et al., 2021; Byregowda et al., 2022; Collavino et al., 2020; Edwards et al., 2015). For instance, root endophytic fungi play fundamental ecological roles in substituting phosphorus strategies in non-mycorrhizal crops or nitrogen availability (Mehta et al., 2019; Sindhu et al., 2024; Sun et al., 2022; Xie et al., 2024). These root endophytes served as a more accurate and effective microbial indicators for evaluating plant resistance to environmental disturbances (Rani et al., 2022; Sun et al., 2021). Although the understanding of microbial community composition in different soil environments has rapidly increased, it remains unclear whether the resistance of root endophytic bacteria and fungi to soil environments influences their beneficial effects on crop yield.

In the natural ecosystem, local microbial communities generally comprise a few dominant species (abundant taxa) and numerous low-abundance species (rare taxa) (Ma et al., 2024; Zheng et al., 2021). The assembly dynamics of abundant and rare taxa in different ecosystems are complex and inconsistent (Gschwend et al., 2021), especially in the context of soil nutrient imbalance in agricultural ecosystems, where they exhibit different distribution patterns and functional characteristics (Jiao et al., 2019a; Ma et al., 2022a; Wu et al., 2023). These differences are attributed to varying microbial survival strategies and substrate preferences (Long and Yao, 2020; Wan et al., 2021). Understanding community assembly process is vital for elucidating the coexistence status among soil microorganisms and preserving species diversity (Chen et al., 2020; Liu et al., 2024). The community assembly process has been categorized into deterministic process, including homogeneous and variable selection, and stochastic process, including homogenizing dispersal, dispersal limitation, and undominated (Chase and Myers, 2011; Stegen et al., 2015). Due to diverse ecological functions and collaborative capabilities of species, the assembly of abundant taxa communities often shows greater stochasticity (Xiong et al., 2020), which underpins the overall stability of the microbial community (Wan et al., 2021). Rare taxa, with high genetic diversity, can adapt to specific environments and play key roles in stabilizing microbial communities and ecosystem functions (Cui et al., 2023; Jia et al., 2022; Ma et al., 2024; Xue et al., 2020; Yu et al., 2024; Zhang et al., 2023). Some key taxa variations of the rare subcommunity have shown ecological potential for mediating microbial interactions, dominating community stability, and contributing to the complexity of symbiotic networks (Jousset et al., 2017; Liang et al., 2020; Xiong et al., 2020). Changes in the proportion and diversity of abundant and rare endophytic subcommunities inevitably affect the stability and ecological function of endophytic communities (Xi et al., 2024; Xiong et al., 2020; Zhang et al., 2024). Thus, understanding the distribution characteristics, assembly processes, and symbiosis patterns of abundant and rare subcommunities is crucial for examining how long-term fertilization affects endophytic microbial diversity and ecological stability.

The use of excessive inorganic nitrogen and phosphorus fertilizers in greenhouse cultivation, driven by yield and economic benefit, is a widespread practice (Wang et al., 2022; Zhang et al., 2017). This practice has led to excessive nutrient accumulation in the soil (Han et al., 2015; Tian et al., 2022) and exacerbated the negative effects on soil microbial community assembly (Bai et al., 2022; Banerjee et al., 2019; Liu et al., 2023b). Traditionally, manure has helped mitigate the harmful effects of chemical fertilizers on microbial communities, thereby contributing to the yield (Zhang et al., 2021). This positive and effective agronomic practice may be key to determining facility crop yield and maintaining agricultural sustainability. Increasing evidence suggests that environmental changes, such as variations in soil nitrogen and phosphorus levels, play a significant role in driving niche variation of root endophytic microbial species and determining the assembly process of root endophytic microbial communities (Bai et al., 2022; Long and Yao, 2020; Sindhu et al., 2024). Appropriate nitrogen levels are conducive to the dominant characteristics of endophytic fungi and endophytic bacterial communities, as well as higher diversity and richness (Liu et al., 2023b; Miranda-Carrazco et al., 2022; Sindhu et al., 2024). The composition and structural changes of rice root endophytic bacterial communities are affected by chemical phosphorus fertilizer (Long and Yao, 2020). Differences in the community structure of root endophytic bacteria and fungi in response to fertilization may be closely related to the host’s nutrient utilization. Therefore, it is essential to identify external environmental factors, particularly nitrogen and phosphorus availability, that influence the assembly of abundant and rare root endophytic subcommunities and their contribution to the yield. Understanding these factors can shed light on the co-evolution of different niche endophytic subcommunities with plants and help develop targeted strategies to manipulate key subcommunities and species to improve crop yields. In this study, we conducted a long-term experiment with nitrogen, phosphorus, and organic manure application in a solar greenhouse. The aims of this study were to (i) identify the relative contributions of the environmental factors in shaping root endophytes and assess how deterministic and stochastic processes influence abundant and rare subcommunities; and (ii) explore the species traits, ecological functions, and co-occurrence patterns of abundant and rare endophytic microbial taxa. We hypothesized the following: (i) The availability of nitrogen and phosphorus in the rhizosphere soil are potential key environmental variables driving stochastic process (dispersal limitation) in endophytic bacterial and fungal assembly, causing structural changes in subcommunities. (ii) Phosphorus-adapting endophytic fungi may occupy niches that complement nitrogen-adaption endophytic bacterial subcommunities, each contributing to main ecological functions. (iii) Rare bacterial subcommunities provide core species that maintain the stability of the endophytic microbial network and complement the functions of abundant bacterial subcommunities, ultimately enhancing crop yield potential.

## 2 Materials and methods

### 2.1 Field site and experimental design

The long-term targeted fertilization experiment was designed in 1996 as a solar greenhouse field at the Shenyang Agricultural University of Liaoning Province, China (41°480 N, 123°250 E). The experimental soil was classified as Glesol (FAO and ISRIC, 1988). The initial basic chemical properties were as follows: pH = 6.75, soil organic matter (SOM) = 24.30 g·kg^−1^, total P (TP) = 1.37 g·kg^−1^, available P (AP) = 70.80 mg·kg^−1^, labile P (LP) = 333.90 mg·kg^−1^, moderately labile P (MLP) = 364.73 mg·kg^−1^, non-labile P (NLP) = 1163.38 mg·kg^−1^, and total N (TN) = 1.16 g·kg^−1^. The fertilization experiment site has a cropping system of continuous tomato cultivation in spring and autumn every year. The fertilization experiment consisted of six treatments arranged in a completely randomized block design (1.5 * 1.0 * 0.8 m^3^ for each pool): (1) CKP0 (unfertilized), (2) CKP1 (chemical phosphorus fertilizer), (3) CNP0 (chemical nitrogen fertilizer), (4) CNP1 (chemical nitrogen plus phosphorus), (5) ONP0 (organic manure), and (6) ONP1 (organic manure plus phosphorus). Each treatment was replicated in three independent cement pools. The fertilizers application rate per year are shown in Supplementary Table S1. Chemical nitrogen fertilizer was applied twice during the tomato whole growing season. Chemical phosphorus fertilizers and organic manure (horse manure obtained 8.69% organic matter, 0.45% total N, and 0.30% total phosphorus) were evenly spread on the surface and incorporated into the plow layer by manual hoeing before planting. The fertilization experiment was conducted in the autumn season of the 26th year from August 2022 to December 2022. Each tomato (“Meisheng”) plant had a single branch after pruning, and there were four clusters of fruits on the branch and five fruits per cluster.

### 2.2 Root and rhizosphere soil sampling

Four rhizosphere soils were randomly collected from each pool and then merged into one rhizosphere soil sample (Bulgarelli et al., 2012). Four tomato root samples were randomly collected from each pool, washed, and merged into one root sample, which was stored in sterilized 1 × PBS (pH = 7.0). All samples were promptly transported to the laboratory. The rhizosphere soil samples were stored at −80°C, while root samples for processed further to prepare for the isolation of root endophytic fungi and bacteria. A total of 2.0 g mixed roots of each sample were surface sterilized in 75% ethanol (1 min), 3.25% sodium hypochlorite (3 min), and 75% ethanol (30 s), followed by three rinses in distilled water (Guo et al., 2000). Root samples were then stored in liquid nitrogen immediately. The key difference was that all solutions used were sterile and the last sterile washed water of each root sample was placed in a PDA medium for 1 week at 28°C to confirm the effectiveness of surface sterilization. A total of 36 experimental samples, including 18 rhizosphere soil samples and 18 root samples (6 treatments × 3 duplicates), were stored at −80°C for subsequent DNA extraction.

### 2.3 Soil chemical parameters determination

Soil pH was measured using 1:2.5 (w/v) soil-to-water suspension. Soil organic carbon (SOC) was determined using the H_2_SO_4_-K_2_Cr_2_O_7_ oxidation capacity method (Mebius, 1960). Available phosphorus (AP) was determined through 0.5 M NaHCO_3_ extraction, while total phosphorus (TP) was measured through H_2_SO_4_-HClO_4_ digestion. Soil phosphorus fractions were prepared and analyzed as reported by Hedley et al. (1982) and Tiessen and Moir (1993). These phosphorus fractions were classified into LP (resin-Pi, NaHCO_3_-Pi, and NaHCO_3_-Po), MLP (NaOH-Pi and NaOH-Po), and NLP (HCl-Pi, concentrated HCl-Pi, concentrated HCl-Po, and residual P) (Maranguit et al., 2017; Shi et al., 2023). Total nitrogen (TN) was quantified using an automatic Kjeldahl distillation–titration method (Cabrera and Beare, 1993). Available nitrogen (AN) was measured using the alkali-hydrolyzed diffusion method, while soil nitrate (NO_3_^−^-N) and ammonium (NH_4_^+^-N) were quantified using a flow-injection autoanalyzer (Skalar San^++^ CFA, Erkelenz, Germany) (Bornemisza et al., 1988).

### 2.4 DNA extraction and high-throughput sequencing

Total DNA was isolated from 0.5 g of pooled root samples (which were created by randomly collecting four surface-sterilized roots and mixing them into a single sample from each pool) using the CretMag^TM^ Plant DNA Mini Kit: CretBiotech, Suzhou, China, following the manufacturer’s instructions. The DNA samples obtained from root samples were amplified using PCR with specific primer sets that included adaptors and barcodes. Fungal ITS1 region amplification was as follows: ITS1F (5′-CTTGGTCATTTAGAGGAAGTAA-3′) and ITS2 (5′- GCTGCGTTCTTCATCGATGC-3′). *16S rDNA* gene V5-V7 region amplification was as follows: 799F (5′- AACMGGATTAGATACCCKG-3′) and 1193R (5′-ACGTCATCCCCACCTTCC-3′) (Liu et al., 2023b). Additionally, to ensure efficiency and accuracy, PCR amplification was performed using TaKaRa Premix Taq® Version 2.0 (TaKaRa Biotechnology Co., Dalian, China), and the reactions took place in the thermocycler PCR system BioRad S1000 (Bio-Rad Laboratory, CA) under the following thermal cycling program for fungi: 95°C/3 min (initial denaturation) followed by 34 cycles of 95°C/20 s, 56°C/20 s, 72°C/30 s, 72°C/5 min (final extension), and lastly held at 12°C. The following thermal cycling program for bacteria is as follows: 98°C/30 s (initial denaturation) followed by 32 cycles of 98°C/10 s, 53°C/20 s, 72°C/30 s, 72°C/2 min (final extension), and lastly hold at 12°C. The PCR mixtures for fungi contain 25 μL of 2 × ES Taq MasterMix(Dye), 2 μL of forward primer (10 μM), 2 μL of reverse primer (10 μM), 50 ng of template DNA, and ddH_2_O added to a final volume of up to 50 μL. The PCR mixtures for bacteria contain 25 μL of Q5, 5 μL of forward primer (10 μM), 5 μL of reverse primer (10 μM), 50 ng of template DNA, and ddH_2_O added to a final volume of up to 50 μL. PCR reactions were performed in triplicate. DNA integrity of isolated DNA was visually inspected using 1.5% agarose gel electrophoresis, and DNA concentration and volume were determined using the NanoDrop2000 (Thermo Fisher Scientific, United States) (For detailed information, refer Supplementary Table S2). During the PCR process, negative controls with water and positive control tests were set. After comparing the concentration of PCR products, the required volume of each sample was calculated based on the principle of equal mass, and the PCR products were mixed accordingly. Target strips were cut and purified using the E.Z.N.A.^®^ Gel Extraction Kit (Omega, United States) and quantified using GeneTools Analysis Software (Version4.03.05.0, SynGene), following the manufacturer’s instructions. The cDNA library was constructed using the NEBNext^®^ Ultra™ II DNA Library Prep Kit for Illumina^®^ (New England Biolabs, United States), according to the manufacturer’s instruction. Purified amplicons were pooled in equimolar and paired-end sequenced by the Shanghai Biotree Biotech Co., Ltd. (Shanghai, China) on the Illumina HiSeq PE250 sequencing platform. The resulting paired sequence reads were merged, trimmed, filtered, and clustered into zero-radius operational taxonomic units using USEARCH (version 0.2.7) with a sequence similarity threshold of 0.97 (Edgar, 2016a; Rognes et al., 2016). The UNITE (ITS) database and the Ribosomal Database Project (RDP) (16S) database were used for taxonomical alignment at a threshold of 0.8 using the SINTAX algorithm (Edgar, 2016b). These databases were used to align multiple sequences, remove annotation for chloroplasts or mitochondria, analyze the phylogenetic relationship between different operational taxonomic units (OTUs), and investigate the differences between the dominant microbial community species across different groups. All of raw reads data are available in the NCBI Sequence Read Archive (SRA) database (Accession Number: PRJNA1182544).

### 2.5 Data analyses

A one-way ANOVA (*p* < 0.05) test was performed to analyze differences in the rhizosphere soil environment factors, yield, and the dominant microbial abundances among different fertilizations using IBM SPSS Statistics (version 22.0, IBM Corporation, Armonk, N.Y., United States) software. The analysis of microbial subcommunity characteristics were performed using R software (version 4.3.0), and some histograms were generated using Origin 2024. Abundant and rare OTUs were defined based on cutoffs above 0.1% and below 0.01% of total sequences, as described in previous studies (Jiao et al., 2017; Jiao and Lu, 2020; Zheng et al., 2021). The “abundant subcommunity” included OTUs with a mean relative abundance >0.1% in root endophytic bacterial and fungal communities across all samples, while the “rare subcommunity” included OTUs with a mean relative abundance <0.01%. The relative abundance of bacterial and fungal subcommunities at the phylum level was constructed using the “*microtable*” package in R (Liu et al., 2020). The Kruskal–Wallis test for *α*-diversity indexes (Shannon and Richness), non-metric multidimensional scaling (NMDS), and analysis of similarities (ANOSIM) were performed using the “*vegan*” package in R (Liu et al., 2015; Oksanen et al., 2012; Yu et al., 2024). The beta diversity was analyzed using the “*betapart*” package in R (Baselga and Orme, 2012). In this study, the Bray–Curtis-based Raup-Crick (RCbray) and the *β*-nearest taxon index (βNTI) were used to calculate the variations in phylogenetic and taxonomic diversity using the “comdistnt” function from the “*picante*” R package (Jiao and Lu, 2020). Quantitatively, the proportion of inferred bacterial and fungal subcommunity assembly ecological processes in root endophytes was analyzed using a phylogenetic bin-based null model analysis (with 999 randomizations), implemented via the “icamp.big” function in R (Ma et al., 2022a; Ning et al., 2020; Wu et al., 2023). Both homogeneous and variable selections were considered as forms of species sorting (deterministic process) (Jiao et al., 2019b). Based on the co-occurrence networks to explore the interrelationships of all OTUs in bacterial and fungal subcommunities, except for the rare and abundant subcommunities, the remaining OTUs were classified as “Others.” Robust correlations were identified using Spearman’s correlation coefficients (*r*) > 0.6 and false discovery rate-corrected *p* values <0.05 (*p* value corrected by FDR multiple test). These metrics were applied to construct random networks of root endophytes and four endophytic subcommunities using the “*igraph*” package in R (Hartman et al., 2018; Jiao et al., 2019b). The topological characteristics of the networks were calculated based on the following metrics: average clustering coefficient, average connectivity, average path distance, and modularity. Furthermore, the keystone species for endophytic subcommunity networks were identified based on the threshold values of Zi (measuring how well a node was connected to other nodes within its module) and Pi (measuring how well a node was connected to nodes between different modules), set at 2.5 and 0.62, respectively (Guimerà and Nunes Amaral, 2005; Lang et al., 2021; Liang et al., 2016; Wu et al., 2023). To identify the key root endophytic subcommunity and taxa predictors for yield, the “*randomForest*” package in R was used, and each predictor was assessed using the “*rfPermute*” package. A Mantel test was conducted to determine the correlation between community composition and rhizosphere soil environment factors. A structural equation model (SEM) was analyzed using IBM SPSS Amos 26 to quantify the direct and indirect effects of the rhizosphere environment factors (such as pH, AN, and MLP) and the *α* and *β* diversities of the bacterial subcommunities on the yield. All variables were standardized using Z transformation (mean = 0, standard deviation = 1) with the ‘scale’ function in the R package. The maximum likelihood method was employed to fit the model (Jin et al., 2020). The standardized total effects (STEs) of the rhizosphere soil nutrients and root endophytic bacterial subcommunities on the yield were calculated.

## 3 Results

### 3.1 Response of the rhizosphere soil physicochemical parameters to long-term fertilization

Long-term fertilization significantly increased the total nutrient content of the rhizosphere soil excluding NH_4_^+^-N (Table 1). The contents of SOC, TN, AN, and NO_3_^−^-N significantly increased in ONP0 and ONP1, by 175%–214%, 71%–84%, 362%–506%, and 956%–1,672%, respectively, compared with CKP0. Different fractions of phosphorus increased with the application of phosphorus and organic manure, with the highest increases in the contents of TP, AP, LP, and NLP observed in ONP1, by 284%, 426%, 330%, and 155%, respectively. The MLP content in ONP1, CKP1, and CNP1 significantly increased by 539%, 587%, and 756%, respectively, compared to CKP0. Soil pH significantly decreased across all treatments compared to CKP0, except in ONP0.

**Table 1:** Effects of long-term fertilization on rhizosphere soil physicochemical parameters.

Treatment	CKP0	CKP1	CNP0	CNP1	ONP0	ONP1
pH (1:2.5 v/w)	7.19 (0.04)a	6.87 (0.04)c	6.84 (0.03)c	6.56 (0.08)d	7.21 (0.02)a	7.00 (0.01)b
SOC (g kg^−1^)	7.85 (0.58)c	8.51 (0.27)c	7.18 (0.40)c	7.58 (0.00)c	21.55 (1.06)b	24.61 (0.53)a
TP (g kg^−1^)	1.12 (0.03)d	3.32 (0.14)b	0.99 (0.01)d	3.30 (0.08)b	1.85 (0.03)c	4.29 (0.15)a
AP (mg kg^−1^)	31.46 (1.51)b	135.94 (2.59)a	23.23 (2.13)b	142.70 (3.58)a	150.11 (13.13)a	165.59 (18.73)a
LP (mg kg^−1^)	247.81 (10.59)e	929.73 (52.35)b	144.42 (14.57)e	801.18 (28.33)c	526.76 (30.63)d	1064.73 (64.31)a
MLP (mg kg^−1^)	163.60 (13.65)c	1125.49 (51.67)b	179.21 (6.61)c	1401.08 (140.42)a	272.29 (8.23)c	1046.20 (64.63)b
NLP (mg kg^−1^)	1132.53 (45.66)c	1906.99 (109.87)b	1000.73 (50.06)c	1671.50 (76.37)b	1518.79 (87.28)b	2884.05 (251.78)a
TN (g kg^−1^)	1.45 (0.15)b	1.29 (0.07)b	1.41 (0.04)b	1.48 (0.04)b	2.49 (0.10)a	2.68 (0.09)a
AN (mg kg^−1^)	9.33 (2.54)c	21.00 (1.75)bc	19.83 (2.33)bc	27.42 (4.21)bc	43.17 (15.47)ab	56.58 (7.10)a
NO_3_^−^-N (mg kg^−1^)	0.80 (0.21)d	1.18 (0.19)d	1.61 (0.52)d	19.51 (0.06)a	8.42 (1.96)c	14.12 (0.56)b
NH_4_^+^-N (mg kg^−1^)	1.43 (0.29)a	1.16 (0.12)a	1.20 (0.13)a	1.87 (0.42)a	1.78 (0.39)a	1.27 (0.09)a

The pH, SOC, TP, AP, LP, MLP, NLP, TN, AN, NO_3_^−^-N, and NO_4_^+^-N refer to rhizosphere soil pH, soil organic carbon, total phosphorus, available phosphorus, labile phosphorus, moderately labile phosphorus, non-labile phosphorus, total nitrogen, available nitrogen, nitrate nitrogen, and ammonium nitrogen, respectively. Lowercase letters denote significant differences based on the Duncan’s one-way analysis of variance among different treatments (*n* = 3, *p* < 0.05).

### 3.2 Response of the endophytic microbial subcommunity characteristics to long-term fertilization

**Figure 1:**
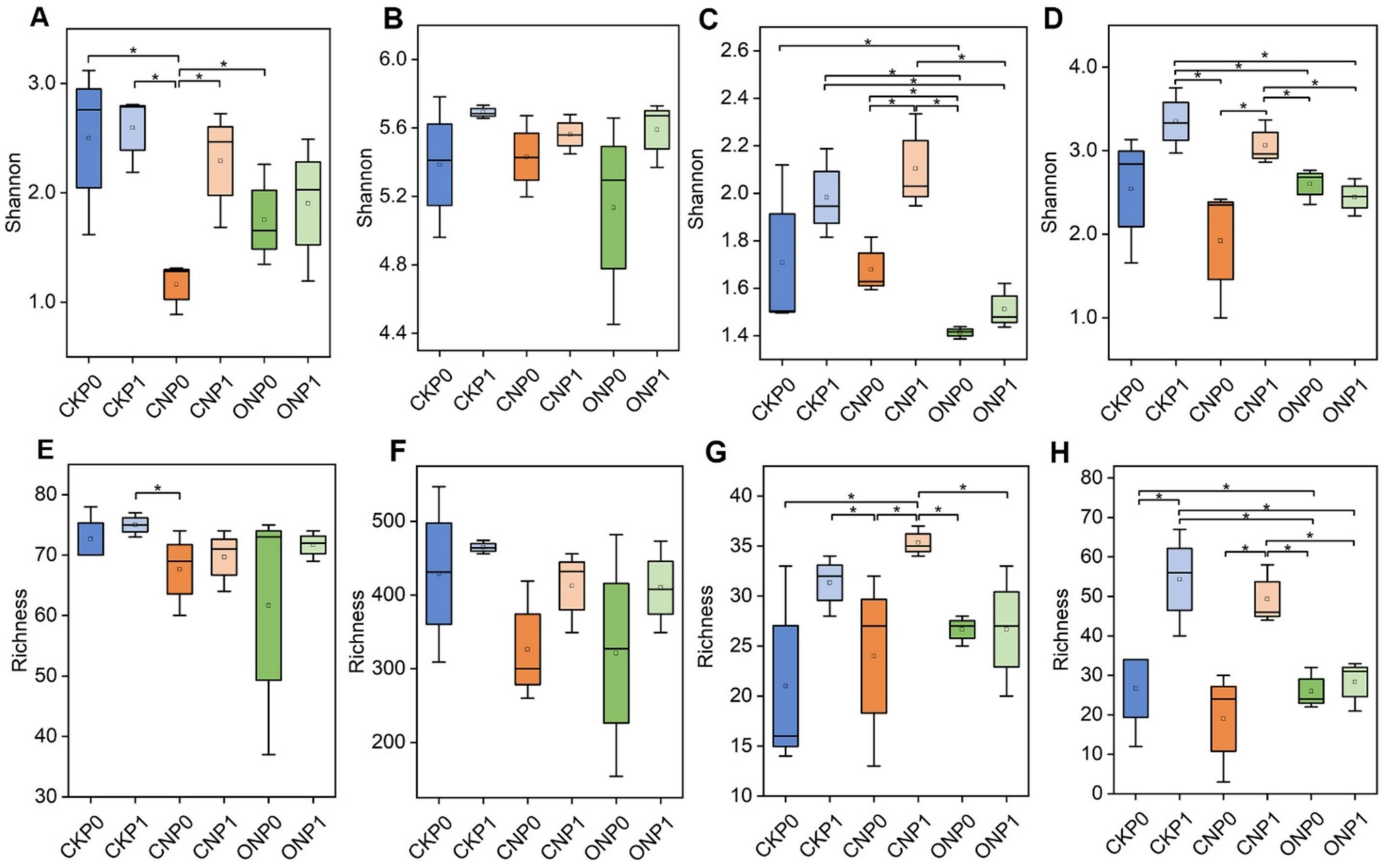
Shannon and richness indexes of root endophytic abundant bacteria **(A,E)**, rare bacteria **(B,F)**, abundant fungi **(C,G)**, and rare fungi **(D,H)** under different treatments. CKP0, Unfertilized; CKP1, Chemical phosphorus applied; CNP0, Chemical nitrogen applied; CNP1, Chemical nitrogen plus phosphorus; ONP0, Organic manure supply nitrogen; ONP1, Organic manure supply nitrogen plus phosphorus. *p < 0.05, as tested by multiple comparisons using the Kruskal–Wallis test, and only significant differences are displayed. The treatment denotations are the same as those in Figure 1.

In tomato roots, the total number of 3,122 bacterial OTUs and 487 fungal OTUs were observed (Supplementary Table S3). A high percentage of OTUs were recognized as rare taxa, constituting rare subcommunities of bacteria and fungi. These rare taxa represented an average of 84.30% and 55.44% of bacterial and fungal OTUs, respectively, but contributed only 6.04% and 0.91% to the average relative abundance in all samples. Conversely, abundant bacterial and fungal taxa constituted a small proportion of OTUs with mean values of 2.85% and 8.00% but accounted for 81.01% and 94.07% of the average relative abundance in all samples, respectively. The rare bacterial subcommunity exhibited higher α-diversity, in terms of both richness and Shannon indexes, compared to the abundant subcommunity under the same treatments. The rare fungal subcommunity showed only a higher Shannon index compared to abundant subcommunity under the same treatments (Figure 1). The Shannon index of abundant bacterial and fungal subcommunities significantly decreased in CNP0 and ONP0 compared to CKP0 (*p* < 0.05; Figures 1A,C). The richness of abundant and rare fungi in CNP1 and ONP0 also significantly decreased compared to CKP0 (*p* < 0.05; Figures 1G,H). There was no significant difference in the Shannon index of rare bacterial and fungal subcommunities or the richness of abundant and rare bacterial subcommunities across other treatments, compared to CKP0 (*p* < 0.05; Figures 1B,D–F).

**Figure 2:**
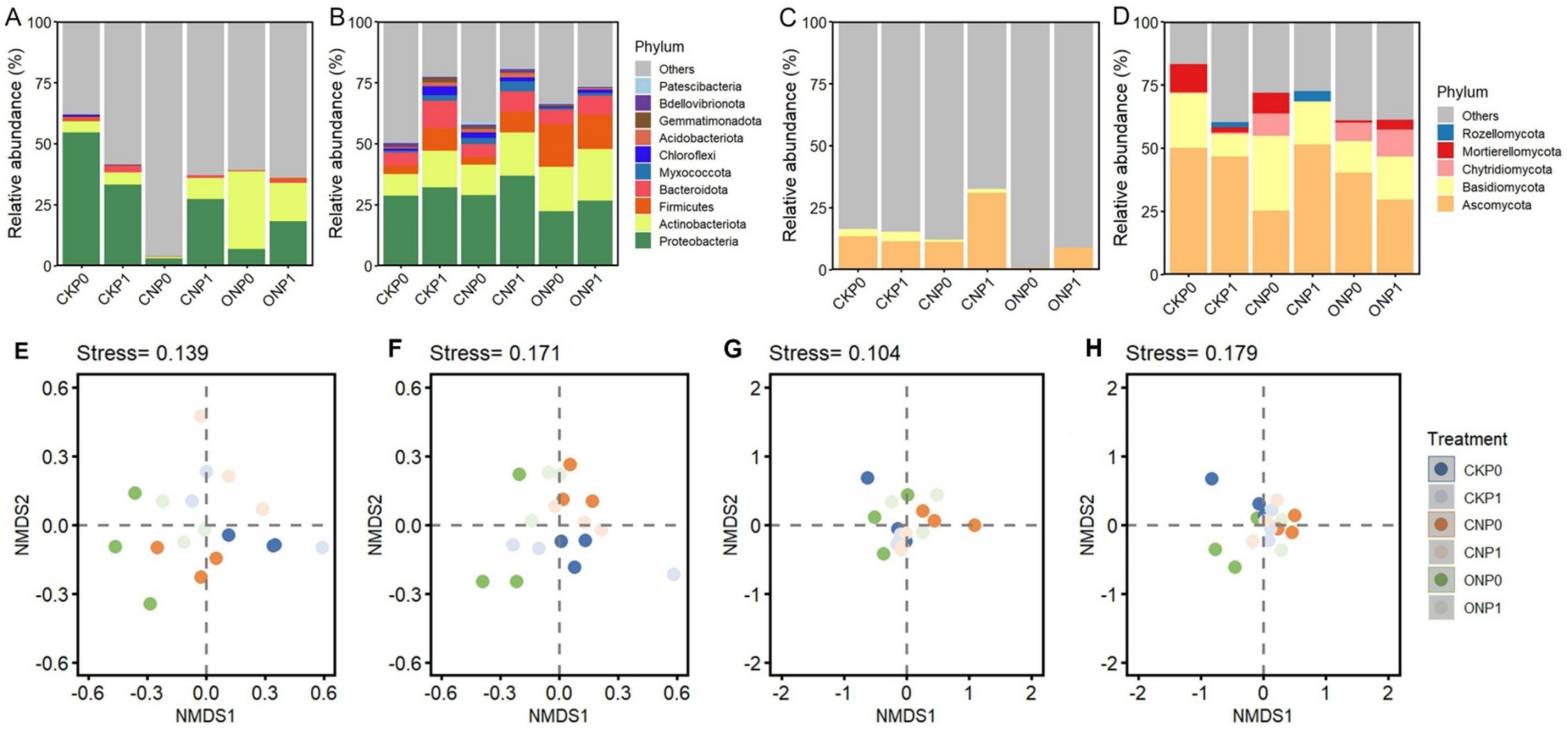
Subcommunity composition at the phylum level and non-metric multidimensional scaling (NMDS) plots of root endophytic abundant bacteria **(A,E)**, rare bacteria **(B,F)**, abundant fungi **(C,G)**, and rare fungi **(D,H)** under different treatments. Unclassified bacterial and fungal OTUs were labeled as “Others.” The treatment denotations are the same as those in Figure 1.

Root endophyte OTUs were classified into 10 bacterial and five fungal phyla, excluding unclassified sequences. The abundant bacterial subcommunity was predominantly Proteobacteria (2.81%–54.50%), whereas the rare bacterial subcommunity was also dominated by Proteobacteria (22.15%–36.75%) (Figures 2A,B). The relative abundance of Proteobacteria in the abundant bacterial subcommunity was significantly decreased by nitrogen application (CNP0 and ONP0) compared to CKP0 (*p* < 0.05; Supplementary Table S4). The relative abundance of Actinobacteriota, Chloroflexi, and Gemmatimonadota in rare bacterial subcommunity was significantly increased in ONP1, CNP1, and CNP1, respectively, compared to CKP0 (*p* < 0.05; Supplementary Table S4). The relative abundance of Acidobacteriota was significantly increased in CKP1, CNP0, and CNP1 compared to CKP0 (*p* < 0.05; Supplementary Table S4). The abundant fungal subcommunity was dominated by Ascomycota (0.8%–13.36%) (Figure 2C), while the rare fungal subcommunity was dominated by Ascomycota (25.23%–51.43%) and Basidiomycota (8.8%–29.63%) (Figure 2D). There was no significant difference in the relative abundance of endophytic fungal phyla among different treatments (*p* < 0.05; Supplementary Table S4). The NMDS analysis of Bray–Curtis distances revealed a distinct separation of all OTUs composition between treatments (beta-diversity). This revealed a clear separation between treatments of the bacterial and fungal subcommunities (Figures 2E–H). The ANOSIM analysis further indicated that the composition of bacterial subcommunity significantly varied with N levels, whereas the composition of the fungal subcommunity significantly varied with P levels rather than among different treatments (*p* ≤ 0.05; Supplementary Table S5).

### 3.3 Community assembly of root endophytic abundant and rare microbial subcommunities

**Figure 3:**
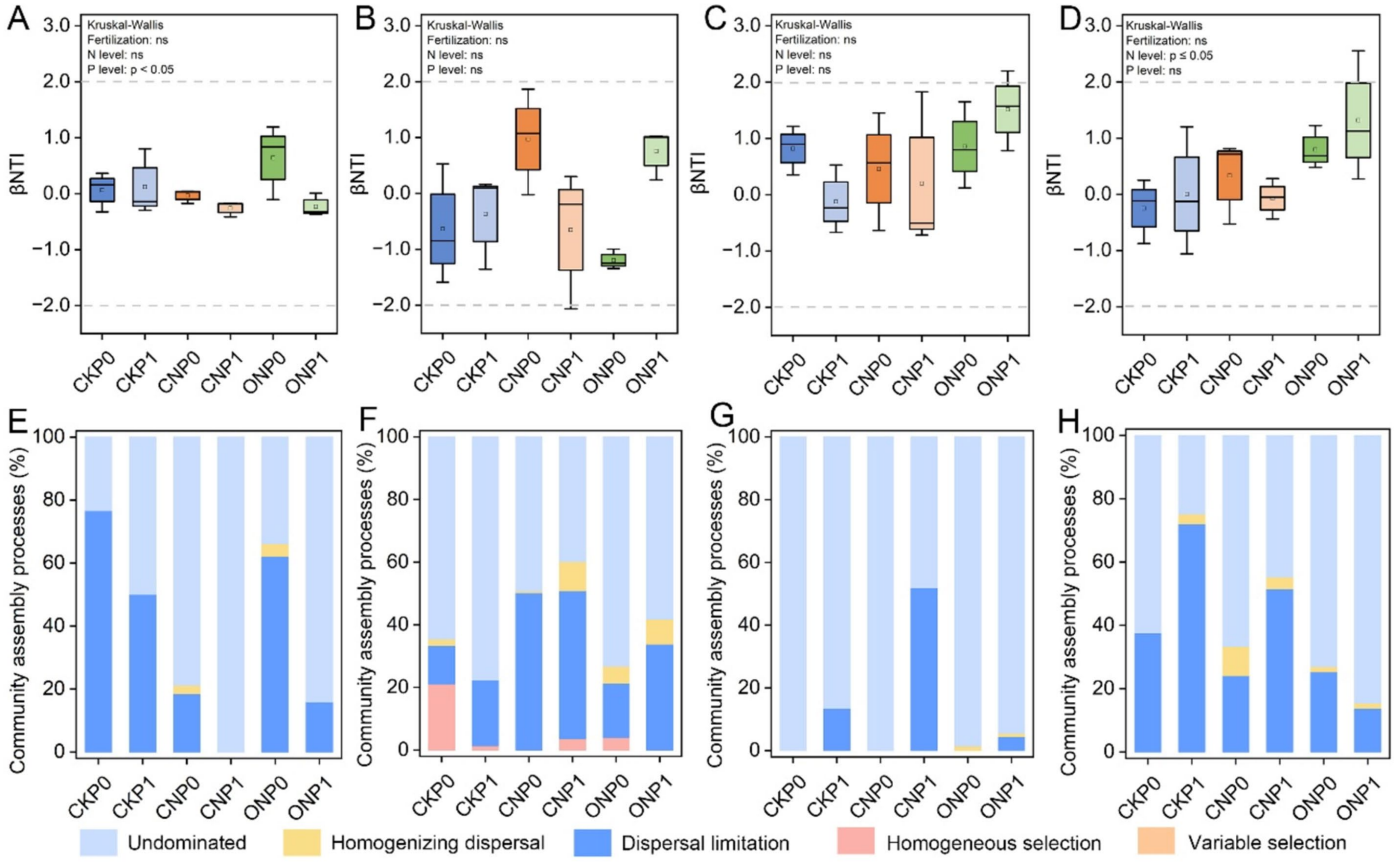
Root endophytic bacterial and fungal subcommunities assembly processes (based on *β*NTI and RCBray). βNTI of root endophytic abundant bacteria **(A)**, rare bacteria **(B)**, abundant fungi **(C)**, and rare fungi **(D)** subcommunities. Proportions of deterministic and stochastic assembly processes in governing root endophytic abundant bacteria **(E)**, rare bacteria **(F)**, abundant fungi **(G)**, and rare fungi **(H)** subcommunities under different treatments.

β-nearest taxon index values were used to quantify the relative contributions of ecological processes in different fertilization treatments (Figures 3A–D). A significant discrepancy in βNTI values of rare fungal and abundant bacterial subcommunities was caused by different N and P levels, respectively (*p* ≤ 0.05). There was no significant discrepancy in βNTI of different microbial subcommunities under different treatments and were all between −2 and + 2. Furthermore, the null model analysis revealed that the ecological assembly of root endophytic abundant and rare subcommunities varied with different treatments. Phosphorus levels mainly governed the abundant bacterial subcommunity through dispersal limitation (23.42%–96.79%), which, and an undominated process (0%–76.58%), which gradually increased (*p* < 0.05; Figures 3A,E). Dispersal limitation (12.26%–50.22%) and undominated (39.82%–77.72%) processes mainly governed the rare bacterial subcommunity (Figure 3F). The abundant fungal subcommunity was entirely governed by stochastic processes, mainly shaped by the undominated process in CKP0, CNP0, and ONP0 and increase dispersal limitation process in P level (CKP1, CNP1, and ONP1) (Figure 3G). The rare fungal subcommunity was mainly governed by nitrogen levels through decreased dispersal limitation (13.73%–72.07%) and increased undominated process (24.82%–84.56%) (Figure 3H). In summary, the assembly of abundant bacterial, abundant fungal, and rare fungal subcommunities was governed by dispersal limitation and undominated processes, while stochastic processes dominated the rare bacterial subcommunity and gradually replaced deterministic processes completely under different treatments (Figure 3).

### 3.4 Keystone species and network topological properties of endophytic microbial subcommunities

**Figure 4:**
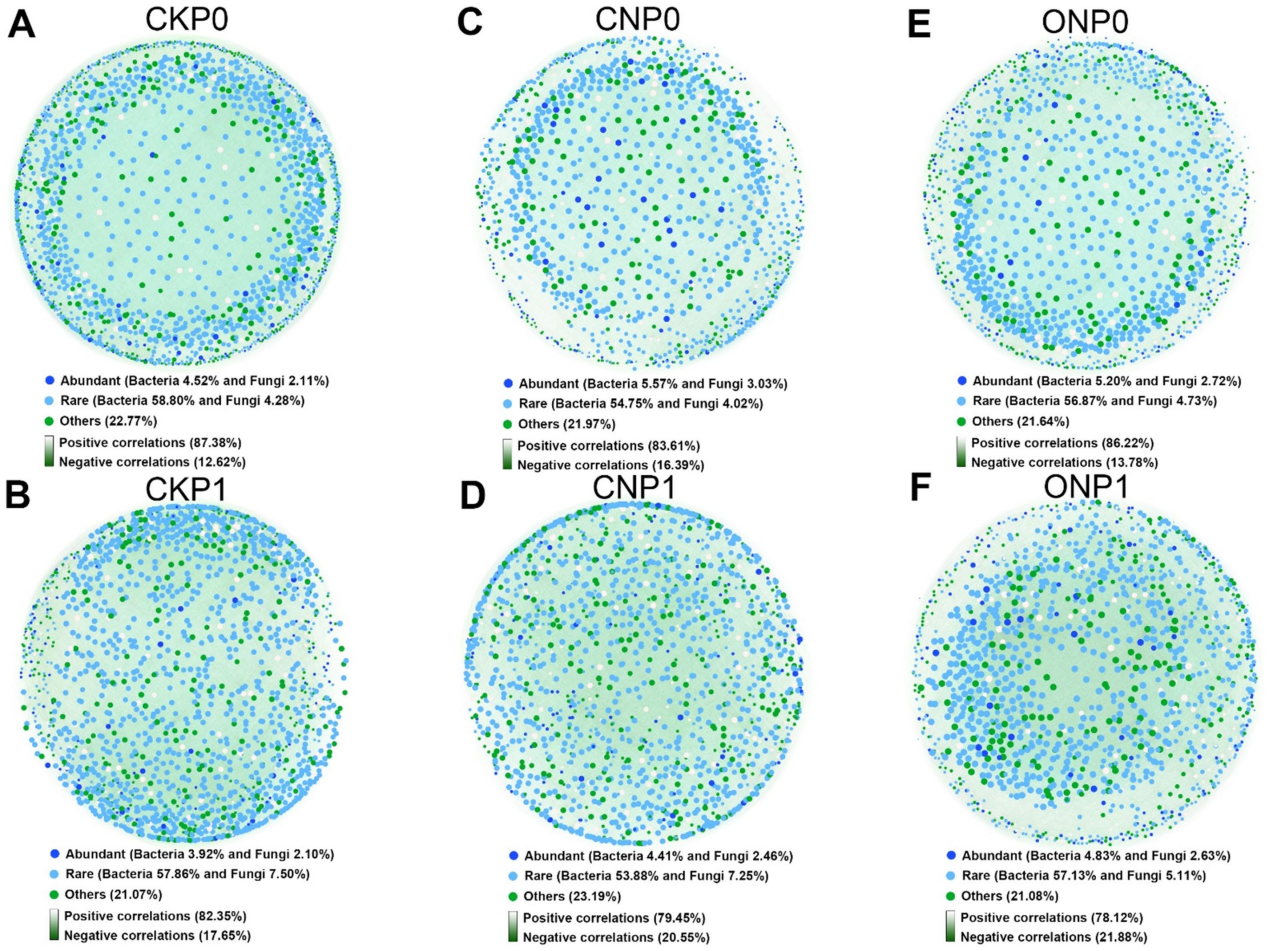
Co-occurrence networks of bacterial and fungal coexistence communities under different fertilizations **(A–F)**. The networks were established by calculating correlations among abundant, rare, and other OTUs.

Co-occurrence networks under different treatments revealed that the endophytic rare subcommunities, with a higher number of nodes, played a more important role than abundant subcommunities (Figures 4A–F). Rare bacterial nodes accounted for 53.88% to 58.80%, whereas abundant bacterial nodes accounted for only 3.92% to 5.57%. Rare fungal nodes accounted for 4.02% to 7.50%, whereas abundant fungal nodes accounted for 2.10% to 3.03%. A co-occurrence network was also constructed for root endophytic abundant and rare subcommunities that conformed to the power-law distribution (Supplementary Figure S2). We observed that the numbers of nodes, edges, and average degree in the root endophytic microbial network decreased with nitrogen application (CNP0, CNP1, ONP0, and ONP1), while the modularity increased and the graph density decreased with phosphorus application (CKP1, CNP1, and ONP1) (Supplementary Figure S1). The observed potential interactions at root endophytes were predominantly positive, accounting for 78.12%–87.38% of the total linkages (Figure 4). The proportion of negative correlations in the root endophytic network of ONP1 was the highest (21.88%) compared to CKP0 (Figure 4F).

**Figure 5:**
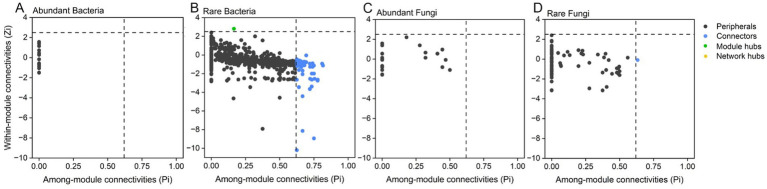
Distributions of network roles by analyzing module features, within-module connectivities (Zi) and among-module connectivities (Pi), in the endophytic abundant bacterial **(A)**, rare bacterial **(B)**, abundant fungal **(C)**, and rare fungal **(D)** co-occurrence networks of root, respectively.

Nitrogen application reduced the modularity and average path length of the abundant bacterial subcommunity network, while increasing the clustering coefficient and average path length and reducing the average degree in the rare bacterial subcommunity network. Phosphorus application increased the modularity and average path length in the network of the abundant fungal subcommunity, while decreasing the clustering coefficient. In the rare fungal subcommunity, phosphorus application increased the clustering coefficient and average degree, but decreased the modularity and average path length (Supplementary Table S6). The topological roles of the individual subcommunity network nodes were shown in a Zi-Pi plot (Figure 5). Peripherals were completely occupied by total nodes in abundant bacterial and abundant fungal subcommunities (Figures 5A,C). We found a total of 63 keystone species in the root endophytic rare bacterial subcommunity network; one was identified as a module hub, while the remaining species served as connectors (Figure 5B). These keystone species play a significant role (2.45%) in the network, and they mainly belong to *Proteobacteria*, *Actinobacteriota*, *Bacteroidota*, *Firmicutes*, *Myxococcota*, *Chloroflex*, *Acidobacteriota*, and *Gemmatimonadota* (Supplementary Table S7). These phyla were highly enriched under ONP1, followed by CKP1. Two keystone species belonging to Ascomycota played a 0.85% role in the rare fungal subcommunity network (Figure 5D; Supplementary Table S7).

### 3.5 Specific subcommunity composition and taxa as predictors of yield under long-term fertilization

**Figure 6:**
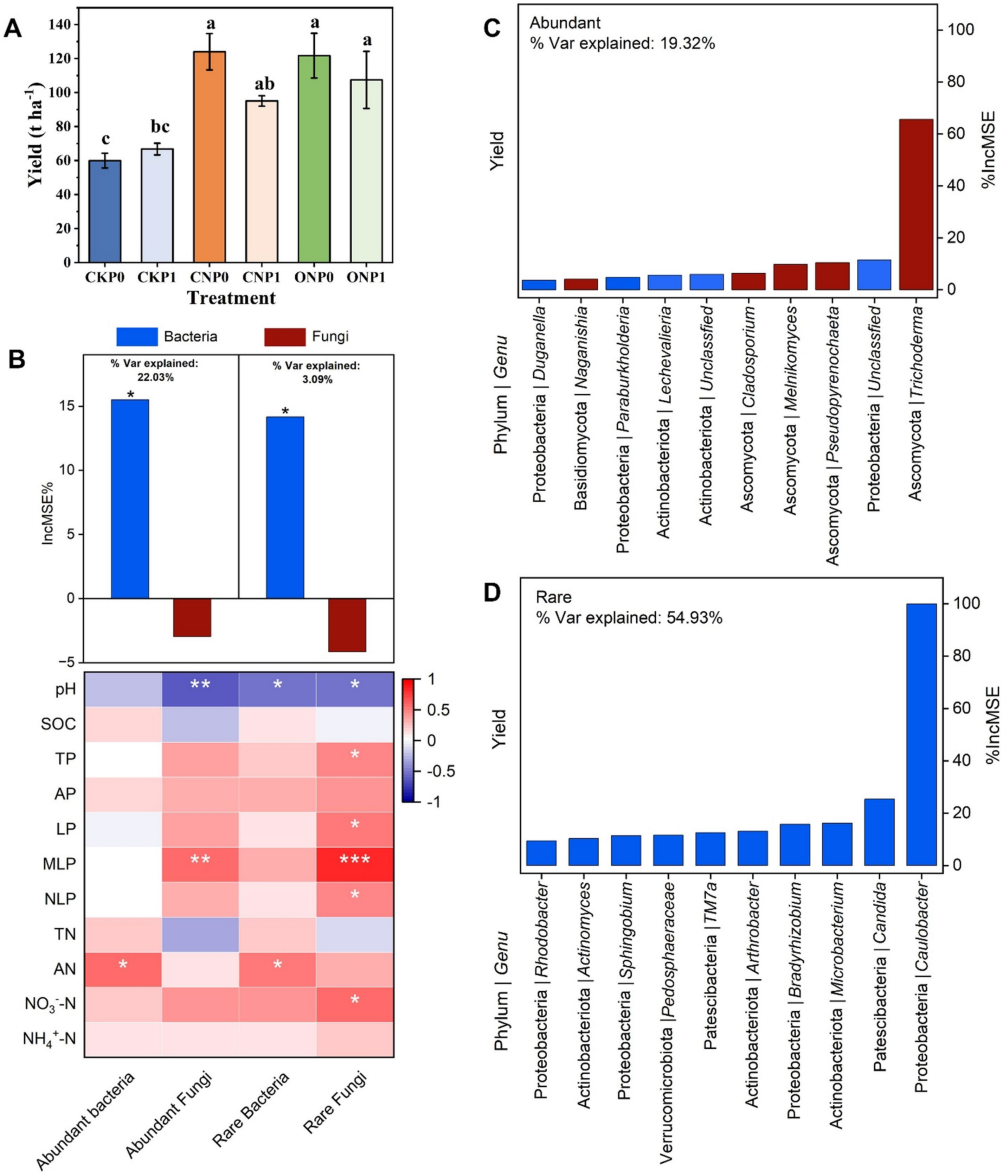
Changes in tomato yield **(A)**. The random forest regression model shows the root endophytic subcommunity structures as drivers of yield. A correlation between the structure of microbial subcommunities and soil nutrients was identified using Spearman’s test **(B)**. The random Forest regression model identifies the top 10 most important taxa of abundant **(C)** and rare **(D)** subcommunities at the genus level, along with their respective phylum, as key drivers of tomato yield. The bars illustrate the increase in mean squared error (%IncMSE) scores for the contribution of root endophytes at the genus level among six fertilization treatments, affecting the tomato yield. Significance levels are denoted with *p < 0.05, **p < 0.01, and ***p < 0.001.

Compared with the CKP0, fertilization treatments significantly increased the yield except CKP1 (*p* < 0.05; Figure 6A). The random forest regression model was used to identify the structure of the root endophytic subcommunity, including abundant bacteria, rare bacteria, abundant fungi, and rare fungi that could predict the yield across different fertilization treatments (Figure 6B). The structure of abundant bacteria and rare bacteria significantly contribute to yield (*p* < 0.05; Figure 6B). Moreover, the structure of the abundant bacterial subcommunity explained 22.03% of yield variation, whereas the rare bacterial subcommunity accounted for 3.09% (Figure 6B). Additionally, the structures of bacterial subcommunities were positively related to AN content (*p* < 0.05; Figure 6B). On the contrary, abundant fungi and rare fungi were positively associated with soil phosphorus especially MLP (*p* < 0.01; Figure 6B), and both of them have no significant positive contribution to yield (Figure 6A). These four subcommunities, except the abundant subcommunity, were negatively correlated to soil pH (*p* < 0.05; Figure 6B). The random forest regression model further predicted the top 10 genera of the rare subcommunity (54.93%) had a greater impact than on yield variation compared to those in the abundant subcommunity (19.32%), indicating that they are more important drivers to yield variation (Figure 6). No key role genus of abundant subcommunity was identified from network, though top 10 abundant genus could predict the yield variation (Figure 6C; Supplementary Table S7). Three keystone species *Arthrobacter*, *Microbacterium*, and *Sphingobium* have been found in the rare microbial subcommunity. These species contribute to increased yield in response to soil nutrient changes and also serve as connectors in the network (Figure 6D; Supplementary Table S7).

**Figure 7:**
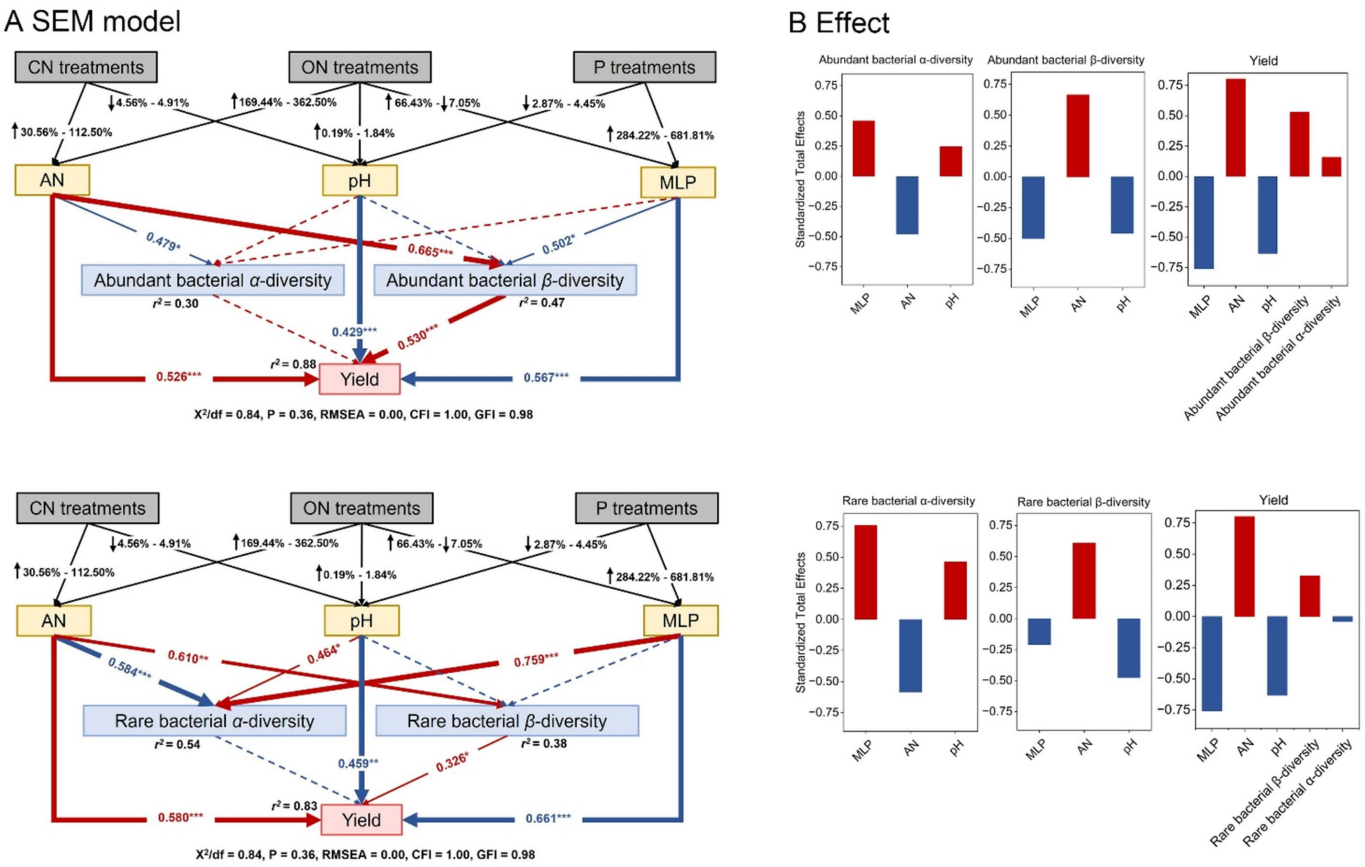
Structural equation model (SEM) illustrating how the root endophytic bacterial subcommunities influenced yield by responding to soil AN, pH, and MLP **(A)**. The total standardized effects of these factors on yield, *α*-diversity (Shannon diversity), and β-diversity are shown **(B)**. Red arrows, black arrows, and dashed arrows indicate the positive significant, negative significant, and nonsignificant relationships between different variables. The width of the arrows indicates the strength of the standardized path coefficient. Adjacent values near the arrows indicate path coefficients. *r*^2^ values indicate the proportion of variance explained by each variable. Significance levels are denoted with *p < 0.05, **p < 0.01, and ***p < 0.001.

Structural equation model was employed to quantify the correlations among key soil characters (AN, pH, and MLP) in response to amounts of N and P fertilizers, the most important root endophytic bacterial subcommunities, and yield (Figure 7). The soil AN, pH, and MLP explained much more variation of the β-diversity (*r*^2^ = 0.47) than α-diversity (*r*^2^ = 0.30) in abundant bacterial subcommunity, but much more variation of α-diversity (*r*^2^ = 0.54) than β-diversity (*r*^2^ = 0.38) in rare bacterial subcommunity. Soil AN had a positive direct and indirect effect by influencing the β-diversity of abundant and rare bacterial subcommunity on yield. It should be noted that the α-diversity of abundant and rare bacterial subcommunities showed no direct effect on yield. The contrasting results were observed for yield, being directly negatively affected by pH and MLP, while through the α-diversity of rare bacteria directly related to MLP (Figure 7A). The soil pH and MLP had a positive effect and AN had a negative effect on the α-diversity of abundant and rare subcommunities, but its effects were contrary on the β-diversity of abundant and rare bacterial subcommunities. The soil AN was found to have the largest standardized positive effect on yield, followed by the β-diversity of abundant and rare bacterial subcommunities, while the contribution of the abundant bacterial β-diversity was greater compared to that of the rare bacterial β-diversity (Figure 7B).

## 4 Discussion

### 4.1 Long-term fertilization affected the construction processes in distribution characters of root endophytes subcommunities

The abundant endophytic bacterial and fungal subcommunities were prevalent in most fertilization treatments with an average relative abundance of 81.01% and 94.07%, respectively. Abundant species may occupy wider niches, competitively utilize more enriched resources, have high growth rates, and effectively adapt to the environment. All of these supported their persistence and their widespread distribution (Jiao and Lu, 2020; Liu et al., 2023a). In addition, the relative abundance of root endophytic rare bacterial and fungal subcommunities that contributed less to the total community abundance was unevenly distributed among different fertilization treatments. The different distribution patterns of root endophytic abundant and rare taxa may be attributed to their distinct life strategies. The rare subcommunities usually exhibited dormancy or low growth rates and competitiveness. Hence, root endophytic microbial subcommunities showed limitations but not extinction in nutrient-enriched environments caused by long-term fertilization (Jia et al., 2018; Jousset et al., 2017). Notably, the rare subcommunities had the highest abundance and diversity compared with abundant subcommunities, which supported the fact that the rare subcommunity was an important contributor to the microbial diversity (du et al., 2020; Li et al., 2021; Lynch and Neufeld, 2015). This trend may be attributed to the fact that a community has a limited capacity and excessive diversity was formed by species competition. Rare taxa can become abundant members of a community in response to favorable environmental conditions (Jia et al., 2022). Few rare species dominated the root samples in our study, but rare bacterial taxa were more susceptible to environmental heterogeneity and with ability to grow fast were affected by nutrient concentration increasing as conditionally rare taxa (Jia et al., 2022; Jiao et al., 2019a). This is possibly due to the selective pressure of resources in the rhizosphere soil environment driving the aggregation of root endophytic rare microbial communities (Liu et al., 2024). It is known that soil environment factors such as carbon, nitrogen, and phosphorus concentrations could across compartments affect root species abundance and interactions (Zhang et al., 2019). These findings suggest that the aggregation process of microbiome with different nutritional strategies can affect the diversity and abundance of endophytic microbial subcommunities (Li et al., 2021; Liu et al., 2023a). Similar evidence has been found in previous studies to explain this change (Dos Santos et al., 2022), where the alpha diversity of endophytic microorganisms increases with the application of fertilizers. However, the α-diversity of fungal subcommunities, especially the α-diversity of the rare fungal subcommunity, was mainly increased by the application of chemical nitrogen and phosphorus fertilizers in our study (*p* < 0.05). Similarly, we found that different N levels and P levels shaped complex rhizosphere soil nutrient heterogeneity that significantly affected the structure of root endophytic bacterial and fungal subcommunities (Ma et al., 2021; Ma et al., 2022b; Zhang et al., 2019). This may have mediated a richer diversity of the rare subcommunity and enhanced their involvement in nutrient cycling processes between environments (Liu et al., 2023a; Shu et al., 2021).

The microbial community assembly processes, which determine the presence and abundance of species, are generally divided into deterministic and stochastic processes (Feng et al., 2018; Jiao et al., 2019b). The null model suggested that the construction of root endophytic bacterial and fungal subcommunities are mainly dominated by stochastic processes especially dispersal limitation (Zhong et al., 2022). More importantly, the assembly process of abundant and rare subcommunities of endophytic bacteria and fungi exhibited different trade-off patterns, reflecting varying relative contributions in response to changes in nitrogen and phosphorus levels (Xing et al., 2023). The proportion of stochastic processes in microbial community assembly can also increase through the functional redundancy generated by biological interactions and spatial and environmental processes (Li and Gao, 2023). Under different fertilization treatments of nitrogen and phosphorus levels, rhizosphere environmental filtration emphasizes the stochastic process of dispersal limitation, which may be involved in determining the development of different sub community systems, which was a very novel finding. Previous studies have revealed that determinism seems to be related to low nutrient levels, and stochasticity increases with nutrient levels (Chase, 2010; Zhou et al., 2014). Therefore, the current nutritional strategy for microbial community assembly processes may be more important than the priority effects shaped by host selection (Li and Gao, 2023). In particular, as the subcommunity rarity increases, dispersal limitation was more conducive to driving the assembly and construction of root endophytic bacterial and fungal subcommunities in enough nitrogen and phosphorus environments, respectively. This validation showed that rare microbial subcommunities were not a random taxonomic cluster; instead, most of these rare subcommunities show restricted distribution or migratory diffusion (Cui et al., 2023; Liu et al., 2015; Long and Yao, 2020). Compared to rare taxa, more individuals in abundant taxa were likely to be involved in dispersal limitation (Liu et al., 2015), such as abundant bacterial subcommunities in our study. From this perspective, abundant taxa are likely to adopt growth yield or resource acquisition strategies that enable them to outcompete other microorganisms for broader niche space and nutrients (Cui et al., 2023; Delgado-Baquerizo et al., 2018; Dong et al., 2021; Jiao and Lu, 2020; Ma et al., 2022a). Consequently, we concluded that long-term fertilization indeed affected the specific niche differentiation of phosphorus-sensitive fungal and nitrogen-sensitive root endophytic bacterial, both abundant and rare subcommunities (Hamonts et al., 2018; Leach et al., 2017; Niu et al., 2017).

The rare fungal subcommunity exhibited a more intense and significant response to phosphorus than the abundant fungal subcommunity, possibly due to the long-life cycle, low community turnover rate, and differences in metabolic activity and diffusion ability of fungi (Jiao and Lu, 2020; Omomowo and Babalola, 2019). Higher nutrient levels increased the importance of stochastic processes (Chase, 2010; Chen et al., 2020), providing insights into the assembly patterns of root endophytic fungal subcommunities promoted by phosphorus. Nitrogen application maintained the control of the dispersal limitation on the enrichment of both abundant and rare bacterial subcommunities, significantly impacting their structure. Previous studies found that short-term high nitrogen levels have a greater effect on the interactions among endophytic fungal communities in maize than on endophytic bacteria (Bai et al., 2022). We found community assembly processes and ANOSIM analysis suggested that endophytic bacteria respond more strongly to nitrogen fertilizer than endophytic fungi. This difference may be partly because nutrient supplementation reduces the competitive advantage of fungi over bacteria (Michalska-Smith et al., 2022) and increases bacterial abundance (Wu et al., 2019), especially in rare bacterial subcommunity. On the other hand, bacteria rely more on fixed sources of nitrogen and carbon than fungi (Schmidt et al., 2014), and organic fertilizers also compensate for the relative lack of carbon when achieving nitrogen supply in this process. These indicated that these subcommunities were formed by more complex assembly processes, especially rare subcommunities, which may also be important metabolic strategies for microbial cells to cope with environmental pressures and be less susceptible to deterministic processes (Fujii et al., 2012; Lennon and Jones, 2011; Nemergut Diana et al., 2013). It is worth noting that stochastic process plays more importance in community assembly, leading to higher β-diversity of microorganisms and higher yield (Chase, 2010). The altered community aggregation characteristics of different subcommunities of root endophytic bacteria and fungi have improved the overall adaptability of microbial communities to their environment, potentially enhancing our understanding of ecosystem sustainability through various means. Rare subcommunities may serve as sensitive biological indicators for regulating their assembly mechanisms and reflecting environmental change risks, closely related to the composition of the entire endophytic microbial community. They may also be a key factor in enhancing the resistance of microbial networks through functional redundancy (Li et al., 2021; Lynch and Neufeld, 2015).

### 4.2 Co-occurrence network of root endophytes subcommunities and keystone species response to long-term fertilization

Network analysis has been identified as a powerful tool for exploring the role of microbial symbiotic interactions in coping with environmental stress (Michalska-Smith et al., 2022; Shu et al., 2021). Fertilizations have affected the network structural characteristics, providing insights into how symbiotic patterns of microbe form different endophytic microbial communities (Liu et al., 2023a) and helping to identify potential keystone taxa in network (Wu et al., 2022; Zhong et al., 2022). Our finding indicated that the frequency, intensity, and distribution patterns of OTU interactions within subcommunities of root endophytic bacteria and fungi vary in response to different fertilization treatments, particularly changed with nitrogen and phosphorus levels. These findings are consistent with previous studies, showing that the cross soil and plant compartments of nutrient concentrations significantly altered overall community dynamics by affecting niche differentiation and colonization of root endophytic bacteria and fungi (Michalska-Smith et al., 2022; Sun et al., 2022; Xu et al., 2022). This niche dispersal selectively recruits microbial subcommunities and formed stable coexistence through root interactions (Ernst et al., 2011). After phosphorus application (CKP1, CNP1, and ONP1), root endophytic microbial communities exhibited higher modularity and lower graph density topological parameters, with an increased proportion of negative cohesion, indicative of a more stable and complex symbiotic pattern. Positive correlations among microbial species have indicated cooperative relationships or niche overlap, while negative correlations have indicated competitive relationships or niche separation (Ernst et al., 2011; Ghoul and Mitri, 2016). Due to higher soil nutrient levels, the highest network complexity and microbial growth diversity have been observed in ONP1, suggesting high nutrient competition among taxa under ONP1 (Deng et al., 2016). This has been verified by the enhanced negative cohesion in the root endophytic symbiotic network under ONP1 (increased by 9.26% and 8.10% compared to CKP0 and ONP0, respectively). Such a complex symbiotic network increased community structure stability, enabling resistance to long-term chemical fertilizer-induced disturbances.

The topological characteristics of the subnetwork indicated that rare subcommunities exhibited more complex and stable symbiotic associations compared to abundant subcommunities (Li et al., 2021). We found that rare bacterial and fungal subcommunities have shown stronger clustering with nitrogen and phosphorus applications, respectively, reflecting the importance of ecological assembly processes and cooperative nutrient metabolism pathways among taxa in the endophytic microbiome (Cui et al., 2023; Fabiańska et al., 2019; Ma et al., 2022b; Sun et al., 2022). The tight associations in the symbiotic network may be the contribution of rare bacterial and rare fungal subcommunities, which were driven by dispersal limitation processes and achieved functional complementarity from different taxonomic compositions. Moreover, under nitrogen level treatments (CN, ON), although the number of nodes and interactions in the subnetwork have decreased, a higher proportion of negative cohesion helped to form more robust competitive relationships and more stable subnetwork, which can be explained by enrichment-dominated and depletion-supplemented patterns (Liu et al., 2024). The complex network structures indicated a more efficient potential for resource and information transmission within the community, allowing it to withstand environmental stress.

Studies have shown that chemical nitrogen fertilizer has significantly affected the structure of root endophytic bacterial communities (Miranda-Carrazco et al., 2022) and reduced the diversity and stability of soil microbial networks (Zhang et al., 2021). Our findings are similar to previous studies, but happened in rare bacterial subnetwork rather than abundant bacterial subnetwork, possibly because the subnetwork structure changes of abundant bacteria have not weakened the core subcommunities or species dominance. Compared to chemical nitrogen fertilizer, long-term organic manure application has likely supported taxonomic groups with similar functions, thereby increasing functional redundancy (Fan et al., 2019). This has a stronger positive effect on maintaining the complexity and stability of the root endophytic bacterial symbiotic network (Zhang et al., 2021), which further enhances the resistance toward the negative effect caused by chemical fertilizers. In general, the root endophytic abundant microbial subcommunities had high stability under different fertilization treatments, which indicated that it could play a dominant role in the symbiotic network. At the same time, the rare subcommunities and their key species may interact with abundant subcommunities under environmental disturbances, forming more stable symbiotic network and performing specific ecological functions (Jiao et al., 2017; Jiao et al., 2019a; Li et al., 2021).

Previous research has also provided evidence that the presence or loss of rare subcommunity and their key species under environmental disturbances can affect the stability of interactions within the entire community, leading to significant structural and functional changes (Cui et al., 2023; Ma et al., 2024). We found that the topological characters of nitrogen-adaption rare bacterial subcommunity tend to align with the structural changes of the entire endophytic microbiome. This further verify that rare endophytic bacterial subcommunity play a significantly more important role than endophytic fungal subcommunities (Liu et al., 2024). The rare bacterial subcommunity contained the most of key species occupying the nodes of module hubs and connectors in the network, and their abundance increases with the application of nitrogen and phosphorus fertilizers. These keystone species can disproportionately and significantly impact ecosystems, with their activity and abundance determining community integrity (Wu et al., 2023). At the phylum level, keystone species in rare bacterial subcommunities belong primarily to *Proteobacteria*, *Actinobacteriota*, *Bacteroidota*, *Firmicutes*, *Myxococcota*, *Chloroflex*, *Acidobacteriota*, *Gemmatimonadota*, and unclassified bacteria.

High nitrogen and phosphorus nutrient concentrations have been unfavorable for the growth of oligotrophic bacteria such as *Acidobacteriota* and *Chloroflexi* (Fierer et al., 2012). Long-term nitrogen and phosphorus fertilizer application has increased the relative abundance of copiotrophic bacteria, including *Alphaproteobacteria*, *Gammaproteobacteria*, *Firmicutes* (Ho et al., 2017), *Bacteroidota*, and *Actinobacteriota* (Dai et al., 2018; Fierer et al., 2012; Sun et al., 2023). Similarly, nitrogen and phosphorus fertilizers have significantly promoted the growth of *Ascomycota*, a hub in the root endophytic fungal subcommunity network, which is considered an r-strategy copiotrophic fungus (Guo et al., 2020). *Ascomycota* is often reported to harbor a high number of genes relevant to stress tolerance and nutrition uptake (e.g., nitrogen immobilization and phosphate transporter) (Egidi et al., 2019). Our results indicated that long-term nitrogen and phosphorus fertilizer applications enriched copiotrophic root endophytic nitrogen-adaption bacteria and phosphorus-adaption fungi, which may possess symbiotic ecological functions (Wu et al., 2023). This enrichment may benefit crop health and high yield in agricultural ecosystems. The most direct reason is that nitrogen and phosphorus fertilizers increased nutrient availability, which may differentially impact communication within bacterial subcommunities in the network, subsequently inducing the transformation of various key groups within the entire microbial community. Therefore, we concluded that long-term balanced fertilization with nitrogen and phosphorus, especially with organic manure and chemical phosphorus fertilizer (ONP1), may enrich more beneficial and important tomato root endophytes, thereby improving microbial network stability and crop yield.

### 4.3 The importance of endophytic bacterial subcommunity structure driven by AN and MLP to yield

Rhizosphere soil nutrient characteristics, particularly nitrogen, phosphorus, and their availability, are potential key factors influencing composition and aggregation of root endophytic microbial community (Liu et al., 2023b; Long and Yao, 2020; Meng et al., 2022; Sindhu et al., 2024). Studies indicated that soil changes are the initial step affecting root endophyte colonization. Although the impact of soil is relatively smaller than that of the host, nitrogen and phosphorus availability still influence endophytic microbial community aggregation and structure (Almario et al., 2017; Edwards et al., 2015). Previous research has shown that different nitrogen levels lead to differences in root endophytic bacteria (Beltran-Garcia et al., 2021). We discovered that different nitrogen supplements caused inconsistent changes in endophytic bacterial community diversity, but structures of endophytic bacteria had similar responses to same rhizosphere soil physicochemical parameters. Long-term different nitrogen fertilizer significantly increased AN content, which showed a significant positive correlation with the structure of both abundant and rare endophytic bacterial subcommunities. Additionally, the reduction in pH was strongly negatively correlated with the structure of rare endophytic bacterial subcommunities, suggesting that pH involved in determining specific endophytic bacterial communities (Lin et al., 2020; Zhang et al., 2024). Previous studies have indicated that endophytic fungal communities are closely related to phosphorus content (Wu et al., 2022). Chemical phosphorus fertilizer provided substantial amounts of labile and moderately labile phosphorus (Maranguit et al., 2017; Shi et al., 2023), and continuous changes in phosphorus availability promote the turnover of endophytic fungal subcommunity structures (Long and Yao, 2020). In non-mycorrhizal crops like tomatoes, it has been found that root endophytic fungi may serve as an alternative strategy for phosphorus utilization, supporting a significant response of endophytic fungal subcommunity to changes in rhizosphere soil MLP content.

Random forest model regression analysis revealed that the most important endophytic subcommunities for predicting crop yield were abundant endophytic bacterial subcommunities, followed by rare bacterial subcommunity, accounting for 22.03% and 3.09% of the variance in predictive factors, respectively. This finding differs from studies on maize-wheat/barley rotation systems (Xiong et al., 2020). In tomato roots affected by long-term fertilization, rare subcommunities structures and species that promote higher network stability may synergistically assist the positive effects of abundant subcommunities with environmental resistance on crop yield. Consequently, the associations between rhizosphere soil nitrogen and phosphorus availability and root endophytic bacterial and fungal subcommunities suggest a pattern of endophytic microbial structural succession, which optimizes microbial communities to influence the relative contributions of different subcommunities to crop yield. Notably, among the top 10 rare subcommunity predictors of yield, three key species were identified that *Arthrobacter*, *Microbacterium*, and *Sphingobium*. Interestingly, both *Arthrobacter* and *Microbacterium* belong to the *Actinobacteriota*, while *Sphingobium* belongs to *Proteobacteria*. These key rare taxa, as plant growth-promoting endophytes (PGPE), have potential to enhance plant resilience to environmental stress and improve crop health and yield, possessing specific functions such as nitrogen fixation and phosphorus solubilization (Machado et al., 2020; Walitang et al., 2017).

## 5 Conclusion

This study provided a systematic understanding of how root endophytic microbial subcommunities, adapted to different ecological niches under long-term fertilization, contribute to maintaining tomato yield. Long-term nitrogen and phosphorus fertilizers increased the proportion of negative cohesion in the root endophytic microbial co-occurrence network, forming ecological niche complementarity between rare bacterial subcommunities and fungal subcommunities. This effect was particularly pronounced in the ONP1 treatment, which led to a more complex, tighter, and stable network structure. The assembly of rare and abundant subcommunities of endophytic bacteria and fungi were dominated by stochastic processes. Nitrogen applications maintained the dispersal limitation ecological process, influencing the assembly process of endophytic rare and abundant bacterial subcommunities, while phosphorus applications controlled the assembly of rare and abundant subcommunities of endophytic fungi by increasing the proportion of the dispersal limitation process. The AN and MLP content in rhizosphere soil significantly affected the community structure changes of nitrogen-adaption root endophytic bacteria and phosphorus-adaption endophytic fungi. The nitrogen-adaption endophytic bacterial subcommunity, particularly the abundant bacterial subcommunity, had a higher explanatory power for tomato yield than the phosphorus-adaption fungal subcommunity. *Arthrobacter*, *Microbacterium*, and *Sphingobium* from the rare subcommunity can potentially serve as beneficial symbiotic endophytes to enhance crop yield. Overall, the application of organic manure combined with chemical phosphorus fertilizer favored the survival strategy of abundant subcommunities and the diversity of rare subcommunities. Ecological complementarity among different subcommunities contributes to forming a more complex and tighter symbiotic network, thereby driving functional redundancy in rare subcommunities potential for high crop yield. The findings have significant implications for sustainable fertilization practices to control root endophytic microbial communities for achieving high crop yield.

## Data Availability

The datasets from this study are available in online repositories, with repository names and accession numbers provided in the article.
