# Supplementary material for: The process of nitrogen-adaptation root endophytic bacterial rather than phosphorus-adaptation fungal subcommunities construction unveiled the tomato yield improvement under long-term fertilization
Source: Front Microbiol. 2025 Jan 17;15:1487323. doi: 10.3389/fmicb.2024.1487323 (PMC11782164; doi:10.3389/fmicb.2024.1487323)
Supplement: Supplementary file 1 [file Data_Sheet_1.pdf]

Table supplements

Table. S1 Fertilizer application rate in different fertilization treatments.

Treatment	Fertilizer Application Amount			Elemental Application Amount			N levels	P levels
	Urea ($kg \cdot ha^{-1}$)	Calcium superphosphate ($kg \cdot ha^{-1}$)	Manure ($kg \cdot ha^{-1}$)	N ($kg \cdot ha^{-1}$)	P_2O_5 ($kg \cdot ha^{-1}$)	K_2O ($kg \cdot ha^{-1}$)		
CKP0	-	-	-	-	-	-	CK	P0
CKP1	-	2397.6	-	-	1149.13	-	CK	P1
CNP0	326	-	-	151.26	-	-	CN	P0
CNP1	326	2397.6	-	151.26	1149.13	-	CN	P1
ONP0	-	-	37500	168.75	112.50	240	ON	P0
ONP1	-	2397.6	37500	168.75	1261.63	240	ON	P1

Table. S2 Summary of data on purity and amount of DNA from each root sample under different fertilization.

Samples	Bacteria V5-V7		Fungi ITS1-2	
	Quality test concentration	Amount	Quality test concentration	Amount
CKP0-1	24.95 ng/3μL	40μL	7.88 ng/3Ml	40μL
CKP0-2	27.45 ng/3μL	40μL	7.88 ng/3μL	40μL
CKP0-3	14.20 ng/3μL	40μL	9.95 ng/3μL	40μL
CKP1-1	14.54 ng/3μL	40μL	9.65 ng/3μL	40μL
CKP1-2	10.68 ng/3μL	40μL	37.53 ng/3μL	40μL
CKP1-3	18.82 ng/3μL	40μL	35.33 ng/3μL	40μL
CNP0-1	31.85 ng/3μL	40μL	14.17 ng/3μL	40μL
CNP0-2	23.46 ng/3μL	40μL	24.57 ng/3μL	40μL
CNP0-3	20.88 ng/3μL	40μL	13.11 ng/3μL	40μL
CNP1-1	22.07 ng/3μL	40μL	17.90 ng/3μL	40μL
CNP1-2	16.17 ng/3μL	40μL	12.39 ng/3μL	40μL
CNP1-3	18.63 ng/3μL	40μL	11.11 ng/3μL	40μL
ONP0-1	24.84 ng/3μL	40μL	36.38 ng/3μL	40μL
ONP0-2	29.92 ng/3μL	40μL	22.19 ng/3μL	40μL
ONP0-3	23.29 ng/3μL	40μL	16.99 ng/3μL	40μL
ONP1-1	31.26 ng/3μL	40μL	13.09 ng/3μL	40μL
ONP1-2	30.12 ng/3μL	40μL	19.24 ng/3μL	40μL
ONP1-3	26.61 ng/3μL	40μL	21.59 ng/3μL	40μL

Table. S3 General description of OTU datasets at 97% sequence similarity level.

OTUs		Numbers of OTU	Shannon	Richness
Bacteria	All	3122	2.54(0.19)	463.61(24.46)
	Abundant	89 (2.85%)		
	Rare	2632 (84.30%)		
Fungi	All	487	1.80(0.08)	61.44(5.13)
	Abundant	39 (8.00%)		
	Rare	270 (55.44%)		

Note: The rare and abundant OTUs of tomato root endophytes. Abundant OTUs were defined as OTUs of bacteria and fungi with a mean relative abundance > 0.1% in all 18 soil samples, respectively. Rare OTUs were defined as OTUs of bacteria and fungi with a mean relative abundance < 0.01% in all 18 samples, respectively.

Table. S4 Comparison of abundant (relative abundance > 0.1%) and rare (relative abundance < 0.01%) microbial taxa at phylum level of tomato root endophytes among different treatments.

Relative abundance (%)	Taxa	CKP0	CKP1	CNP0	CNP1	ONP0	ONP1
Abundant bacteria	Proteobacteria	54.50(20.00)a	33.04(9.65)ab	2.81(1.01)b	27.15(11.76)ab	6.72(3.45)b	18.10(9.73)ab
Rare bacteria	Actinobacteriota	9.04(1.94)b	14.99(2.99)ab	12.59(3.29)ab	17.77(1.78)ab	18.22(5.62)ab	21.01(1.86)a
	Acidobacteriota	0.48(0.20)b	1.44(0.09)a	1.38(0.43)a	2.04(0.19)a	0.04(0.04)b	0.53(0.33)b
	Chloroflexi	1.00(0.50)b	3.62(0.93)a	2.31(0.43)ab	1.56(0.50)b	0.37(0.20)b	1.34(0.81)b
	Gemmatimonadota	0.39(0.14)b	1.81(0.05)a	0.87(0.42)ab	0.37(0.19)b	0.65(0.31)ab	0.42(0.33)b

Note: Only endophytic microbial taxa with significant differences are listed in the table. Values are treatment means (standard error) followed by different letters in each column denoting significant differences among treatments at $p < 0.05$.

Table. S5 Dissimilarity test showing differences of root endophytic abundant and rare subcommunities based on Bray-Curtis distance across nitrogen and phosphorus fertilizer levels and treatments.

	ANOSIM							
	Abundant Bacteria		Rare Bacteria		Abundant Fungi		Rare Fungi	
	R	*p*	R	*p*	R	*p*	R	*p*
**NP fertilizer level**								
N level	0.151	**0.022***	0.131	**0.041***	0.052	0.121	0.020	0.337
P level	< 0.000	0.468	0.019	0.326	0.076	**0.050***	0.079	**0.017***
**Treatments**								
CKP0 vs. CKP1	0.185	0.200	0.222	0.400	0.222	0.200	0.148	0.200
CKP0 vs. CNP0	0.444	0.100	0.630	0.100	0.000	0.600	0.294	0.100
CKP0 vs. CNP1	0.185	0.200	0.444	0.100	0.111	0.400	0.481	0.100
CKP0 vs. ONP0	0.074	0.400	< 0.000	0.800	0.333	0.100	0.222	0.200
CKP0 vs. ONP1	0.444	0.100	0.556	0.100	0.111	0.400	0.222	0.200
CNP0 vs. CNP1	0.482	0.100	0.815	0.100	0.111	0.400	0.815	0.100
ONP0 vs. ONP1	< 0.000	0.900	0.222	0.100	0.185	0.300	< 0.000	0.700

Note: ANOSIM, analysis of similarity. Dissimilarities of OTU level in root endophytic abundant and rare subcommunities were calculated based on the Bray-Curtis distance. Nitrogen fertilizer level represents the subcommunities differences among CK (CKP0 and CKP1), CN (CNP0 and CNP1), and ON (ONP0 ONP1). Phosphorus fertilizer level represents the subcommunities differences among P0 (CKP0, CNP0 and ONP0), P1 (CKP1, CNP1 and ONP1). P-values were corrected by false discovery rate (FDR) in BH method. The red label represents significant values ($p \leqslant 0.05$).

Table. S6 Topological properties of networks in root endophytic microbial subcommunities under long-term different nitrogen and phosphorus fertilizer levels.

Topological properties		Nodes	Edges	Clustering coefficient	Modularity	Average path length	Average degree	Network diameter	Graph density
**Abundant bacteria**	CK	78	226	0.517	0.582	4.355	5.795	12	0.075
	CN	77	298	0.549	0.458	3.257	7.740	9	0.102
	ON	80	269	0.476	0.546	3.510	6.725	8	0.085
**Rare bacteria**	CK	631	13039	0.467	0.639	0.638	41.328	5	0.066
	CN	489	8060	0.515	0.637	2.848	32.965	5	0.068
	ON	532	9724	0.506	0.688	2.836	36.556	5	0.069
**Abundant fungi**	P0	35	90	0.608	0.470	2.154	5.143	6	0.151
	P1	34	82	0.513	0.506	3.098	4.824	8	0.146
**Rare fungi**	P0	109	254	0.015	0.692	6.422	4.661	15	0.043
	P1	217	986	0.022	0.666	4.093	9.088	10	0.042

Note: Nitrogen and phosphorus levels rather than different fertilization treatments affected the structure of endophytic bacterial and fungal subcommunity. Then the influence of nitrogen and phosphorus on the topological properties of subnetwork are analyzed. Nitrogen fertilizer level represents the subcommunities differences among CK (CKP0 and CKP1), CN (CNP0 and CNP1), and ON (ONP0 ONP1). Phosphorus fertilizer level represents the subcommunities differences among P0 (CKP0, CNP0 and ONP0), P1 (CKP1, CNP1 and ONP1).

Table. S7 The information of root endophytic rare bacterial and fungal keystone species in co-occurrence network of subcommunities.

	OTU_ID	Phylum	Class	Order	Family	Genus
Rare bacteria	OTU1821	Acidobacteriota	Holophagae	Holophagales	Holophagaceae	Unclassified
	OTU1560	Actinobacteriota	Actinobacteria	Micrococcales	Microbacteriaceae	Unclassified
	OTU2814	Actinobacteriota	Actinobacteria	Pseudonocardiales	Pseudonocardiaceae	Unclassified
	OTU1816	Actinobacteriota	Actinobacteria	Propionibacteriales	Unclassified	Unclassified
	OTU933	Actinobacteriota	Actinobacteria	Micrococcales	Micrococcaceae	Arthrobacter
	OTU218	Actinobacteriota	Actinobacteria	Streptosporangiales	Thermomonosporaceae	Thermostaphylospora
	OTU292	Actinobacteriota	Actinobacteria	Propionibacteriales	Nocardioidaceae	Nocardioides
	OTU1680	Actinobacteriota	Actinobacteria	Euzebyales	Euzebyaceae	Unclassified
	OTU46	Actinobacteriota	Actinobacteria	Streptosporangiales	Streptosporangiaceae	Nonomuraea
	OTU2671	Actinobacteriota	Actinobacteria	Propionibacteriales	Nocardioidaceae	Unclassified
	OTU311	Actinobacteriota	Actinobacteria	Streptomycetales	Streptomycetaceae	Streptomyces
	OTU1901	Actinobacteriota	Actinobacteria	Micrococcales	Microbacteriaceae	Microbacterium
	OTU2078	Actinobacteriota	Thermoleophilia	Solirubrobacterales	67-14	67-14
	OTU1732	Actinobacteriota	Actinobacteria	Micromonosporales	Micromonosporaceae	Actinoplanes
	OTU2067	Actinobacteriota	Thermoleophilia	Solirubrobacterales	Unclassified	Unclassified
	OTU2060	Actinobacteriota	Thermoleophilia	Solirubrobacterales	67-14	67-14
	OTU2560	Bacteroidota	Bacteroidia	Flavobacteriales	Flavobacteriaceae	Lutimonas
	OTU2822	Bacteroidota	Bacteroidia	Flavobacteriales	Flavobacteriaceae	Flavobacterium
	OTU2567	Bacteroidota	Bacteroidia	Cytophagales	Microscillaceae	Unclassified
	OTU1934	Bacteroidota	Bacteroidia	Chitinophagales	Chitinophagaceae	Chitinophaga
	OTU2546	Bacteroidota	Bacteroidia	Cytophagales	Microscillaceae	Unclassified
	OTU2833	Bacteroidota	Bacteroidia	Flavobacteriales	Weeksellaceae	Chryseobacterium
	OTU534	Bacteroidota	Bacteroidia	Cytophagales	Microscillaceae	Unclassified
	OTU2865	Chloroflexi	Dehalococcoidia	S085	S085	S085
	OTU2887	Chloroflexi	Chloroflexia	Chloroflexales	Roseiflexaceae	Unclassified
	OTU1657	Firmicutes	Bacilli	Lactobacillales	Streptococcaceae	Streptococcus

OTU1890	Firmicutes	Bacilli	Paenibacillales	Paenibacillaceae	Unclassified
OTU887	Firmicutes	Bacilli	Paenibacillales	Paenibacillaceae	Unclassified
OTU1514	Firmicutes	Bacilli	Bacillales	Bacillaceae	Oceanobacillus
OTU919	Firmicutes	Bacilli	Paenibacillales	Paenibacillaceae	Paenibacillus
OTU911	Firmicutes	Bacilli	Bacillales	Bacillaceae	Bacillus
OTU200	Gemmatimonadota	Gemmatimonadetes	Gemmatimonadales	Gemmatimonadaceae	Unclassified
OTU1279	Myxococcota	Polyangia	Haliangiales	Haliangiaceae	Haliangium
OTU1035	Myxococcota	Polyangia	Polyangiales	Sandaracinaceae	Unclassified
OTU1001	Myxococcota	Polyangia	Haliangiales	Haliangiaceae	Haliangium
OTU1250	Proteobacteria	Alphaproteobacteria	Rhodospirillales	Unclassified	Unclassified
OTU658	Proteobacteria	Alphaproteobacteria	Sphingomonadales	Sphingomonadaceae	Sphingobium
OTU766	Proteobacteria	Alphaproteobacteria	Sphingomonadales	Sphingomonadaceae	Sphingopyxis
OTU1990	Proteobacteria	Gammaproteobacteria	Pseudomonadales	Moraxellaceae	Acinetobacter
OTU2494	Proteobacteria	Gammaproteobacteria	Burkholderiales	Comamonadaceae	Unclassified
OTU454	Proteobacteria	Alphaproteobacteria	Rhizobiales	Rhizobiaceae	Rhizobium
OTU2597	Proteobacteria	Alphaproteobacteria	Caulobacterales	Caulobacteraceae	Asticcacaulis
OTU2021	Proteobacteria	Gammaproteobacteria	Unclassified	Unclassified	Unclassified
OTU729	Proteobacteria	Alphaproteobacteria	Acetobacterales	Acetobacteraceae	Rhodovastum
OTU440	Proteobacteria	Alphaproteobacteria	Rhizobiales	Rhizobiaceae	Rhizobium
OTU802	Proteobacteria	Gammaproteobacteria	Pseudomonadales	Moraxellaceae	Unclassified
OTU2518	Proteobacteria	Unclassified	Unclassified	Unclassified	Unclassified
OTU1517	Proteobacteria	Alphaproteobacteria	Sphingomonadales	Sphingomonadaceae	Sphingobium
OTU2093	Proteobacteria	Alphaproteobacteria	Rhizobiales	Stappiaceae	Labrenzia
OTU545	Proteobacteria	Alphaproteobacteria	Rhizobiales	Rhizobiaceae	Unclassified
OTU1905	Proteobacteria	Gammaproteobacteria	Burkholderiales	Comamonadaceae	Comamonas
OTU3300	Unclassified	Unclassified	Unclassified	Unclassified	Unclassified
OTU1573	Unclassified	Unclassified	Unclassified	Unclassified	Unclassified
OTU3078	Unclassified	Unclassified	Unclassified	Unclassified	Unclassified
OTU3293	Unclassified	Unclassified	Unclassified	Unclassified	Unclassified
OTU3106	Unclassified	Unclassified	Unclassified	Unclassified	Unclassified
OTU1968	Unclassified	Unclassified	Unclassified	Unclassified	Unclassified

	OTU105	Unclassified	Unclassified	Unclassified	Unclassified	Unclassified
	OTU3348	Unclassified	Unclassified	Unclassified	Unclassified	Unclassified
	OTU165	Unclassified	Unclassified	Unclassified	Unclassified	Unclassified
	OTU2992	Unclassified	Unclassified	Unclassified	Unclassified	Unclassified
	OTU3067	Unclassified	Unclassified	Unclassified	Unclassified	Unclassified
	OTU158	Unclassified	Unclassified	Unclassified	Unclassified	Unclassified
Rare	OTU308	Ascomycota	Sordariomycetes	Unclassified	Unclassified	Unclassified
fungi	OTU429	Ascomycota	Sordariomycetes	Hypocreales	Cylindriaceae	Cylindrium

Fig supplements

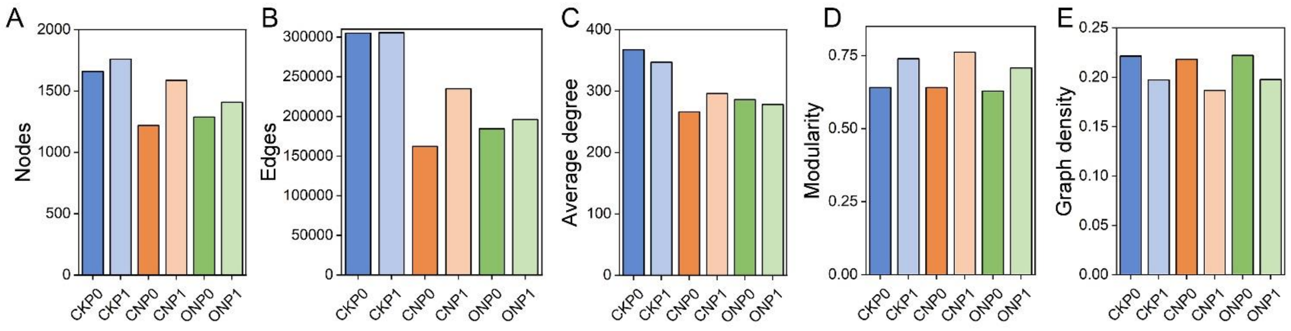

Fig. S1 Topological properties of networks in root endophytic microbial subcommunities under different treatments.

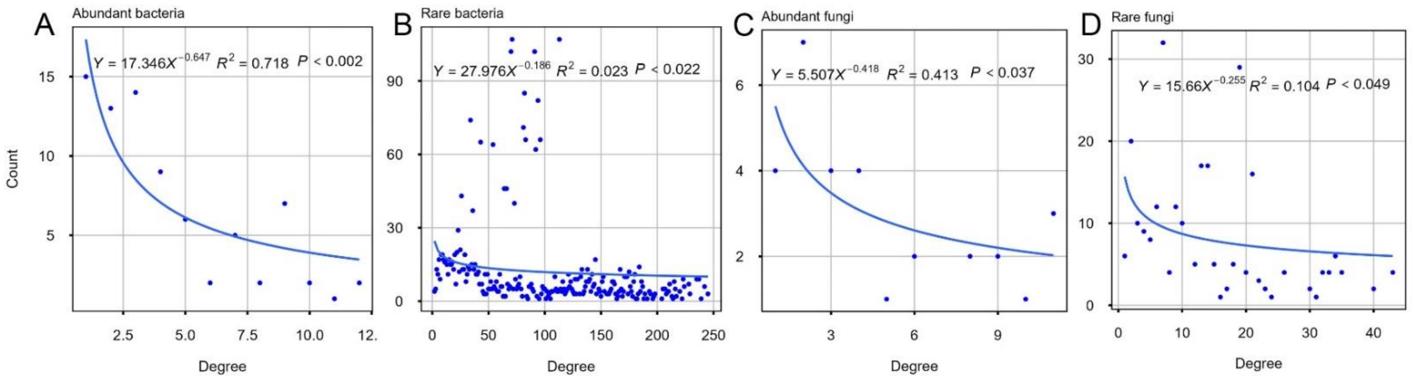

Fig. S2 Network topological properties of the degree distribution pattern of four subcommunities in root endophytic bacteria and fungi.

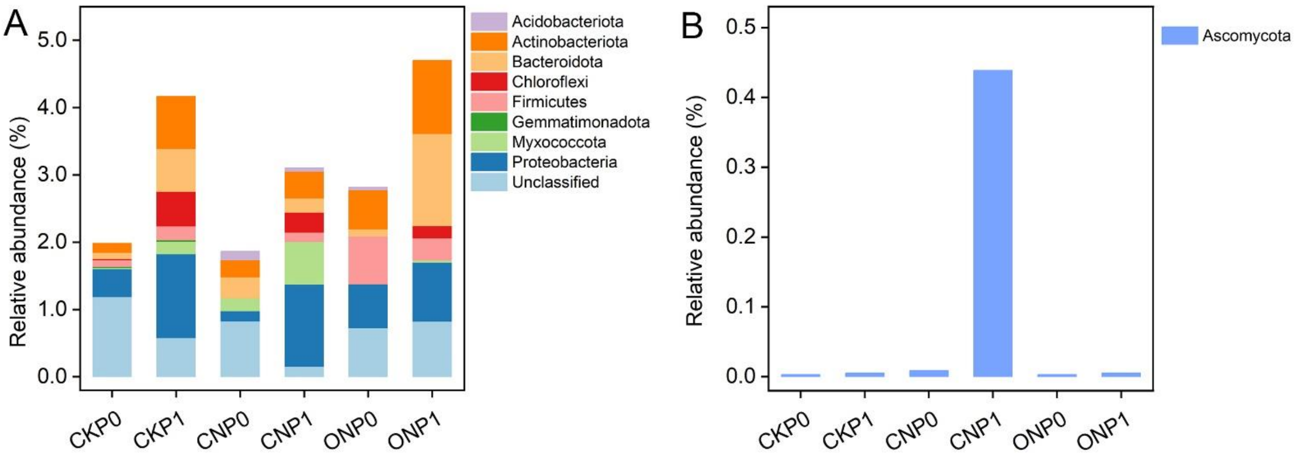

Fig. S3 The relative abundance of keystone species at the phylum level in endophytic rare bacterial and rare fungal subcommunities.
